# Has resourcing of non‐governmental harm‐reduction organizations in Ukraine improved HIV prevention and treatment outcomes for people who inject drugs? Findings from multiple bio‐behavioural surveys

**DOI:** 10.1002/jia2.25608

**Published:** 2020-08-26

**Authors:** Adam Trickey, Nadiya Semchuk, Tetiana Saliuk, Yana Sazonova, Olga Varetska, Josephine G Walker, Aaron G Lim, Jack Stone, Peter Vickerman

**Affiliations:** ^1^ Population Health Sciences University of Bristol Bristol United Kingdom; ^2^ Alliance for Public Health Kiev Ukraine

**Keywords:** Ukraine, harm reduction, prevention, HIV, hepatitis C virus, NGO

## Abstract

**Introduction:**

People who inject drugs (PWID) in Ukraine have high prevalences of HIV and hepatitis C (HCV). Since the turn of the century, various organizations have funded non‐governmental organizations (NGOs) in Ukraine to provide PWID with needles and syringes, condoms, HIV and HCV testing, and improve linkage to opioid agonist therapy (OAT) and HIV treatment. We investigated whether contact with these NGOs was associated with improved HIV prevention and treatment outcomes among PWID.

**Methods:**

Five rounds of respondent‐driven sampled integrated bio‐behavioural survey data (2009 [N = 3962], 2011 [N = 9069], 2013 [N = 9502], 2015 [N = 9405], and 2017 [N = 10076]) among PWID in Ukraine (including HIV/HCV testing and questionnaires) were analysed using mixed‐effect logistic regression models (mixed‐effects: city, year). These regression models assessed associations between being an NGO client and various behavioural, OAT, HIV testing and HIV treatment outcomes, adjusting for demographic characteristics (age, gender, lifetime imprisonment, registration in a drug abuse clinic, education level). We also assessed associations between being an NGO client and being HIV positive or HCV positive, likewise adjusting for demographic characteristics (as above).

**Results:**

NGO clients were more likely to have received HIV testing ever (adjusted odds ratio [aOR] 5.37, 95% confidence interval [95% CI]: 4.97 to 5.80) or in the last year (aOR 3.37, 95% CI: 3.20 to 3.54), to have used condoms at last sexual intercourse (aOR 1.37, 95% CI: 1.30 to 1.44) and sterile needles at last injection (aOR 1.37, 95% CI: 1.20 to 1.56), to be currently (aOR 4.19, 95% CI: 3.48 to 5.05) or ever (aOR 2.52, 95% CI: 2.32 to 2.74) on OAT, and to have received syringes (aOR 109.89, 95% CI: 99.26 to 121.66) or condoms (aOR 54.39, 95% CI: 50.17 to 58.96) in the last year. PWID who were HIV positive (aOR 1.40, 95% CI: 1.33 to 1.48) or HCV positive (aOR 1.57, 95% CI: 1.49 to 1.65) were more likely to have contact with NGOs, with HIV positive PWID in contact with NGOs being more likely to be registered at AIDS centres (aOR 2.34, 95% CI: 1.88 to 2.92) and to be on antiretroviral therapy (aOR 1.60, 95% CI: 1.40 to 1.83).

**Conclusions:**

Contact with PWID targeted NGOs in Ukraine is associated with consistently better preventive, HIV testing and HIV treatment outcomes, suggesting a beneficial impact of harm reduction NGO programming.

## 1 INTRODUCTION

Globally, there are an estimated 15 million people who inject drugs (PWID), amongst whom there is a high prevalence of HIV (17.8%) and exposure to hepatitis C virus (HCV) infection (52.3%), primarily due to bloodborne transmission through unsterile injections [1, 2]. Estimates of the global disease burden of HIV due to injecting drug use (IDU) are low (4% globally [3]), but high in some regions, whereas for HCV this figure is around 40% for both disease burden [3] and incidence [4]. Eastern Europe and Central Asia is the only region where the number of HIV infections and HIV‐related deaths are increasing [5], with this region also having the highest general population prevalence of HCV [6]. Ukraine has the second biggest epidemic of both HIV and HCV in the region and in Europe overall [6, 7, 8]. In Ukraine, the prevalence of IDU among adults (approximately 1%) is higher than the global average (approximately 0.3%) [1], with an estimated 50% and 80% of the HIV and HCV burdens, respectively, being due to IDU [3].

Due to their high prevalence and incidence of HIV and HCV, PWID, both globally and in Ukraine, require targeted interventions to reduce transmission levels and to provide disease care and treatment. These measures include needle and syringe provision (NSP), opioid agonist therapy (OAT), condom distribution, curative treatment for HCV, and antiretroviral therapy (ART) for HIV. Evidence shows high coverage of NSP and OAT are effective at reducing HIV and HCV acquisition among PWID [9, 10]. Curative treatment for HCV can also reduce HCV transmission [11], whereas ART can effectively halt HIV transmission by reducing viral loads to undetectable levels [12]. Initiating HIV positive PWID onto ART requires them to be diagnosed and linked to care, which requires a high coverage of testing.

There has been progress combating HIV in Ukraine, with UNAIDS reporting AIDS‐related deaths halving from 14,000 to 7,900 over 2010 to 2015 [13]. Most HIV treatment and prevention funding currently comes from the Global Fund to fight AIDS, tuberculosis, and malaria, with 547 million US dollars (USD) disbursed since 2003. Most funds have gone to non‐governmental organizations (NGOs) such as the Alliance for Public Health (APH) and 100% Life [14]. These NGOs aim to reduce the epidemics of HIV, hepatitis and tuberculosis among the most vulnerable populations in Ukraine, including PWID, men who have sex with men and sex workers. In cooperation with other organizations, they distribute condoms and needles/syringes, provide HIV and HCV testing and counselling, and encourage linkage to ART and OAT. The Ukrainian government provides ART in AIDS centres and runs OAT programmes. Recently, global funders have reduced funds to middle‐income country settings based on the assumption that their governments can and should fund interventions for HIV, HCV and tuberculosis. Because of this, funding for Ukraine from the Global Fund is decreasing, with the latest grant for Ukraine supporting the transition of prevention services to the government [15]. There are concerns that this could reduce funding for HIV, exacerbated by a recent economic crisis and war with Russia [16], raising concerns of possible reductions in services for key populations.

APH has undertaken seven repeated nationwide cross‐sectional integrated bio‐behavioural surveys (IBBS) over 2004 to 2017. The primary aim of this study was to use data from surveys undertaken between 2009 to 2017 to determine whether being a client of an NGO is associated with improved HIV and HCV prevention and treatment outcomes and related injecting‐ or sexual‐risk behaviours among PWID in Ukraine.

## 2 METHODS

### 2.1 Setting and sample

Data came from five nationwide IBBS among PWID in Ukraine covering the years 2009, 2011, 2013, 2015 and 2017 [17, 18, 19, 20]. Full details of the sampling methodology are given in each survey report (the 2017 report is not yet available, but methods are similar). In brief, the IBBS used respondent‐driven sampling (RDS) to recruit PWID. Eligible participants had injected drugs in the last 30 days, were aged ≥14 years and resided in a participating city. They needed to give consent to being surveyed, provide a dried blood spot sample, and agree to HIV and/or HCV testing. A person could partake in multiple survey rounds. The number of initial respondents (seeds) varied between 2 and 6 for different cities and years depending on the planned sample size. These seeds had to be aged under 26 and be HIV negative. They were selected to cover a range of parameters regarding gender, age, drug use and harm reduction contact. Table S1 presents the number of overall participants in each city for each survey round, and their regions. There were 31 cities included, which covered all of Ukraine’s 27 regions, and so should be representative of Ukraine. Fifteen cities were included in all five rounds, whereas 11 were included in four rounds. The surveys were carried out in various locations, mostly rented office blocks, AIDS centres and sometimes offices of organizations providing services to PWID. The surveys were often carried out by NGOs.

### 2.2 Measures

Questions were asked about demographic characteristics, injecting and sexual behaviours, harm reduction intervention contact and outcomes, recent contact and duration of contact with NGOs, HIV/HCV testing history, self‐reported HIV/HCV diagnosis status and HIV treatment uptake. The questions included in each survey were similar across rounds with some minor differences, and, in some years, particular questions were added or excluded. Our analyses focus on questions that are comparable across all rounds. Data regarding whether someone is a client of an NGO was obtained through self‐report, using the question: “Are you a client of any non‐governmental organization (have a card or individual code), that provides prevention services for injection drug users?”. Further details of the questions can be found in Table S2.

HIV and HCV testing were also performed in each survey (except HCV testing in 2009). Rapid tests for HIV and HCV were used to determine a respondent’s status.

### 2.3 Analyses

We chose not to use RDS‐weights in the main analysis due to a lack of consensus around their use for regression models [21], particularly when RDS surveys across multiple sites are combined. However, we present RDS‐weighted characteristics for comparison in sensitivity analyses.

### 2.4 Comparing PWID by NGO client status

Tests for differences in behaviours and preventive outcomes by current NGO client status were assessed by either χ^2^ tests or t‐tests, depending on whether the variable was binary or continuous.

### 2.5 Characteristics associated with being an NGO client

We tested for general non‐intervention related characteristics associated with being an NGO client (vs. not), using mixed‐effect logistic regression with year and city as crossed random effects.

In unadjusted and adjusted analyses, we investigated whether testing HIV positive (vs. negative), testing HCV antibody positive (vs. negative), age (years), female sex (vs. male), having ever been imprisoned (vs. not), being registered in a drug abuse clinic (vs. not) and education level (categorical: see supplementary materials) were associated with being an NGO client. For 2009, information on imprisonment was unavailable so data from this year were not included in these models. In a sensitivity analysis, we removed imprisonment and HCV from the model and included the 2009 survey data.

### 2.6 Associations between being an NGO client and intervention‐related outcomes

To assess for associations between NGO client status and various intervention‐related outcomes around HIV/HCV and their transmission, we used mixed‐effect logistic regression models with year and city as crossed mixed‐effects. In mixed‐effects models the adjusted odds ratios should be interpreted as holding all other variables in the model constant, as well as the random effects for city and year. The use of mixed‐effects models was to account for variation in levels of service provision and epidemiological characteristics between cities and years (and differences between years within cities), without explicitly modelling this. Unadjusted and adjusted associations of the outcomes with NGO client status, age, female sex, having ever been imprisoned, being registered in a drug abuse clinic and education level were assessed.

### 2.7 NGO client duration

Mixed‐effect logistic regression models with the same structure were used to assess trends between duration of NGO client status (assessed as a continuous variable in years among those with known duration) and various risk behaviour and intervention outcomes. These models were also adjusted for age, except the model with age as the outcome (a mixed‐effect linear regression model). NGO client duration data were unavailable for 2009 so this survey was omitted from these analyses.

### 2.8 Use of services by PWID

Data from the earliest (2011) and most recent survey (2017) with self‐reported use of HIV services were compared over the two years. The outcomes compared were the number of PWID testing HIV+, the number of these that self‐reported HIV+, the number reporting being registered at an AIDS centre, and the number reporting that they receive ART. Tests for differences in the use of services by whether PWID were NGO clients or not were assessed using χ^2^ tests.

### 2.9 Trends over time

Trend tests for variables across multiple survey years were performed using logistic or linear regression, depending on whether the outcome variable was binary or continuous, with cluster‐robust standard errors being used for clustering by city.

### 2.10 Ethical approval

The surveys underwent examination by the Committee of Medical Ethics at the Institute of Epidemiology and Infectious Diseases of the Ukrainian Academy of Medical Sciences. Informed consent was obtained from all study participants.

## 3 RESULTS

The number of PWID surveyed in each IBBS were 3963 (2009), 9069 (2011), 9502 (2013), 9405 (2015) and 10,076 (2017). Table  shows characteristics and behaviours of the PWID surveyed in each year and tests for trends. Across all surveys, around one‐third of PWID surveyed self‐reported as NGO clients, which was stable over the years. A sensitivity analysis using RDS‐weighted estimates (Table S3) gave similar results.

**Table 1:** Behaviours and preventive outcomes among PWID across each survey year, with a test for trends across years^a^

Variable	2009 (N = 3962)	2011 (N = 9069)	2013 (N = 9502)	2015 (N = 9405)	2017 (N = 10076)	Overall	Trend test^a^ coefficient (95% CI)	*p*‐value
% NGO client	36.6%	32.6%	38.4%	26.3%	33.7%	33.1%	−0.05 (−0.17, 0.08)	0.466
Mean NGO client duration (years)	NA	2.4	2.6	3	4.6	3.2	0.73 (0.59, 0.87)	<0.001
% Female	23.4%	25.6%	22.5%	19.7%	17.8%	21.5%	−0.12 (−0.16, −0.07)	<0.001
% Completed secondary education	81.3%	84.5%	78.9%	81.4%	82.9%	81.9%	−0.00 (−0.08, 0.08)	0.964
Mean age (years)	30.7	32.8	33.4	34.3	35.5	33.7	1.05 (0.77, 1.34)	<0.001
Mean age of first injection (years)	19.4	20	18.7	20.1	20.3	20	0.18 (0.01, 0.34)	0.034
Mean injecting duration (years)	11.3	12.8	13.7	14.1	15.2	13.7	0.88 (0.60, 1.16)	<0.001
Mean injections last month	NA	25.9	16.8	19.1	21.2	20.7	−1.08 (−2.69, 0.53)	0.179
% Primary drug is opioid	80.1%	77.6%	82.5%	81.0%	82.5%	81.9%	0.06 (−0.05, 0.18)	0.293
% Overdosed last year	12.9%	8.1%	6.3%	5.9%	5.2%	7.0%	−0.22 (−0.34, −0.10)	<0.001
% Ever on OAT	NA	7.9%	12.2%	13.7%	10.4%	11.0%	0.08 (−0.02, 0.19)	0.115
% Currently on OAT	NA	NA	NA	5.0%	4.6%	4.8%	−0.08 (−0.53, 0.36)	0.713
% Registered in drug abuse clinic	34.7%	33.7%	35.5%	30.8%	31.1%	32.9%	−0.05 (−0.13, 0.03)	0.238
% Currently homeless	NA	NA	0.5%	0.3%	0.3%	0.3%	−0.31 (−0.67, 0.04)	0.084
% Ever imprisoned	NA	33.4%	34.9%	39.7%	41.5%	37.5%	0.13 (0.06, 0.19)	<0.001
% Imprisoned in last year	NA	4.7%	5.2%	10.4%	5.3%	6.4%	0.11 (0.04, 0.18)	0.003
% Last needle used was sterile	90.8%	95.7%	96.6%	94.6%	96.8%	95.5%	0.17 (0.03, 0.32)	0.020
% Needles used last month that were unsterile	NA	1.7%	1.4%	0.8%	0.9%	1.2%	−0.03 (−0.05, −0.01)	0.006
% Using a pre‐filled syringe last month	60.6%	59.1%	57.0%	48.0%	36.9%	50.9%	−0.27 (−0.35, −0.18)	<0.001
% Using condom last intercourse (among those who had had sex)	54.0%	54.5%	54.7%	49.9%	46.7%	51.6%	−0.09 (−0.15, −0.03)	0.003
% Received syringes last year	51.6%	51.3%	52.5%	37.4%	39.1%	45.6%	−0.18 (−0.30, −0.06)	0.004
% Buying syringes last month	NA	67.1%	70.0%	82.6%	84.7%	76.3%	0.37 (0.25, 0.49)	<0.001
% Received or bought syringes^b^	NA	95.3%	97.4%	95.4%	97.1%	96.3%	0.10 (−0.10, 0.30)	0.349
% Received condoms last year	47.8%	50.2%	50.2%	35.3%	37.3%	43.5%	−0.17 (−0.28, −0.06)	0.002
% Buying condoms last month	NA	22.5%	23.0%	22.1%	18.8%	21.6%	−0.07 (−0.14, −0.00)	0.038
% Received or bought condoms^b^	NA	65.7%	66.7%	52.5%	52.5%	59.2%	−0.22 (−0.34, −0.11)	<0.001
% HIV tested ever	50.0%	69.2%	76.0%	71.6%	78.5%	71.7%	0.23 (0.15, 0.32)	<0.001
% HIV tested last year	NA	39.0%	41.8%	38.6%	39.4%	39.7%	−0.01 (−0.10, 0.08)	0.859
% Aware of HIV‐positive status (among those testing HIV positive)	39.2%	46.5%	57.9%	43.6%	54.4%	49.2%	0.09 (−0.03, 0.21)	0.137
% Registered in an AIDS centre (of those self‐reported HIV positive)	86.9%	80.8%	86.4%	90.1%	88.7%	86.6%	0.09 (−0.08, 0.26)	0.300
% on ART (of those registered in an AIDS centre)	NA	30.3%	54.4%	60.3%	67.5%	53.8%	0.51 (0.40, 0.62)	<0.001
% HIV positive	23.8%	22.7%	18.1%	22.0%	22.3%	21.5%	−0.01 (−0.08, 0.07)	0.875
% HIV positive of PWID aged <25 years	9.0%	8.1%	4.3%	4.3%	3.7%	6.1%	−0.29 (−0.49, −0.09)	0.004
% HCV antibody positive	NA	37.7%	56.6%	54.3%	63.8%	53.4%	0.31 (0.19, 0.42)	<0.001
% HCV antibody positive of PWID aged <25 years	NA	20.3%	32.7%	23.8%	28.0%	26.0%	0.10 (−0.04, 0.23)	0.176

ART, antiretroviral therapy; CI, confidence interval; HCV, hepatitis C virus; NGO, non‐governmental organization; OAT, opioid agonist therapy; PWID, people who inject drugs.

^a^ Trend test coefficient produced using logistic or linear regression modelling (depending on variable type) with survey year as an independent variable and clustering of standard errors by city

^b^ composite variable created from the above two variables.

### 3.1 Comparing PWID by NGO client status

Table 2 compares the characteristics and behaviours of NGO/non‐NGO clients across survey years and overall. All variables analysed showed differences between these two groups. In these analyses, NGO clients were more likely to be female, older, and to have completed secondary education. They tended to inject more frequently, were more likely to primarily inject opioids and to have ever been imprisoned, but less likely to have been imprisoned in the last year. NGO clients were more likely to be registered in a drug abuse clinic and to have ever been or currently on OAT. They were more likely to have received syringes or condoms in the last year and conversely were less likely to have bought syringes or condoms in the last month. NGO clients were more likely to have either received or bought syringes in the last year, which was also the case for condoms. NGO clients were also more likely to have used condoms for their last sexual intercourse and were more likely to have used a sterile needle for their last injection. For HIV, they were more likely to have ever been tested in the last year or ever. A greater proportion of NGO clients self‐reported as HIV+ (20.4% vs. 7.0%) and tested HIV+ (29.2% vs. 17.8%) or HCV+ (64.5% vs. 48.1%). Of those self‐reporting HIV+, a greater proportion of NGO clients were registered at an AIDS centre and, of these, a greater proportion reported receiving ART. Mean monthly income in 2017 (when data were available) for NGO clients was lower than for non‐NGO clients, 4,663 vs. 5,219 Ukrainian hryvnia (approximately 190.46 vs. approximately 213.17 USD on 23 January 2020). Table S4 shows categorized education‐level stratified by year and NGO client status.

**Table 2:** Behaviours and preventive outcomes among PWID across each survey year and combined across survey years, stratified by whether they are NGO clients or not and tested for differences^a^

Variable	Non‐NGO					Overall	NGO					Overall	Test^a^
	2009	2011	2013	2015	2017		2009	2011	2013	2015	2017		
	N = 2516	N = 6115	N = 5853	N = 6932	N = 6679	N = 28095	N = 1446	N = 2954	N = 3649	N = 2474	N = 3397	N = 13920	
% Female	22.0%	24.0%	20.0%	17.9%	16.4%	19.7%	25.8%	28.8%	26.4%	24.7%	20.6%	25.1%	<0.001
% Completed secondary education	82.3%	83.1%	80.3%	80.1%	82.3%	81.5%	79.7%	87.2%	76.8%	85.1%	84.1%	82.6%	0.008
Mean age (years)	29.8	32.7	33.1	33.7	34.9	33.3	32.1	33	33.8	35.8	36.8	34.5	<0.001
Mean age of first injection (years)	19.7	20.3	20.4	20.5	20.7	20.4	18.8	19.3	18.6	19.2	19.5	19.1	<0.001
Mean injecting duration (years)	10.1	12.4	12.7	13.2	14.2	12.9	13.3	13.7	15.2	16.7	17.3	15.4	<0.001
Mean injections last month	NA	22.4	15.2	18.7	20.1	19.2	NA	33.2	19.4	20.1	23.5	23.9	<0.001
% Primary drug is opioid	77.5%	76.0%	82.0%	79.5%	79.2%	79.0%	84.5%	80.9%	83.3%	85.1%	88.8%	84.6%	<0.001
% Overdosed last year	10.1%	6.7%	5.5%	5.6%	5.3%	6.2%	16.5%	10.9%	7.6%	7.0%	5.0%	8.5%	<0.001
% Ever on OAT	NA	4.2%	7.7%	9.2%	4.7%	6.5%	NA	15.5%	19.5%	26.0%	21.4%	20.4%	<0.001
% Currently on OAT	NA	NA	NA	2.8%	1.2%	2.0%	NA	NA	NA	11.3%	11.4%	11.4%	<0.001
% Registered in drug abuse clinic	24.8%	26.9%	26.5%	23.5%	21.7%	24.5%	51.9%	48.0%	50.0%	51.4%	49.5%	49.9%	<0.001
% Currently homeless	NA	NA	0.6%	0.3%	0.3%	0.4%	NA	NA	0.3%	0.3%	0.2%	0.2%	0.044
% Ever imprisoned	NA	29.0%	29.9%	36.4%	38.6%	33.7%	NA	42.6%	42.9%	48.9%	47.1%	45.2%	<0.001
% Imprisoned in last year	NA	4.9%	5.5%	10.0%	6.0%	6.7%	NA	4.4%	4.7%	11.5%	3.9%	5.8%	<0.001
% Last needle used was sterile	88.9%	95.2%	96.0%	93.9%	96.5%	94.8%	94.5%	96.8%	97.4%	96.7%	97.4%	96.8%	<0.001
% Needles used last month that were unsterile	NA	0.3	0.2	0.1	0.2	1.4%	NA	0.4	0.1	0.1	0.1	0.9%	<0.001
% Using a pre‐filled syringe last month	59.2%	58.5%	56.7%	48.2%	37.0%	50.5%	62.9%	60.3%	57.4%	47.5%	36.5%	51.7%	<0.001
% Using condom last intercourse^b^	48.7%	52.4%	51.0%	47.9%	44.6%	48.8%	62.9%	58.8%	60.5%	55.2%	51.0%	57.2%	<0.001
% Received syringes last year	24.8%	29.8%	24.5%	17.3%	11.4%	20.8%	98.1%	95.9%	97.5%	93.6%	93.5%	95.5%	<0.001
% Buying syringes last month	NA	80.9%	86.7%	91.2%	94.0%	88.4%	NA	38.5%	42.6%	58.5%	66.5%	51.3%	<0.001
% Received or bought syringes^c^	NA	93.4%	96.0%	93.9%	95.9%	94.8%	NA	99.2%	99.7%	99.6%	99.5%	99.5%	<0.001
% Received condoms last year	23.6%	30.0%	23.0%	15.7%	11.0%	19.9%	89.9%	91.9%	93.7%	90.5%	88.9%	91.2%	<0.001
% Buying condoms last month	NA	29.7%	31.7%	26.7%	24.1%	27.9%	NA	7.7%	9.0%	9.2%	8.5%	8.6%	<0.001
% Received or bought condoms^c^	NA	52.6%	49.2%	38.4%	33.2%	42.9%	NA	92.8%	94.7%	91.9%	90.5%	92.5%	<0.001
% HIV tested ever	35.4%	59.0%	65.8%	63.9%	70.1%	62.1%	75.5%	90.4%	92.3%	93.3%	95.0%	91.0%	<0.001
% HIV tested last year	NA	28.2%	32.3%	30.0%	27.2%	29.5%	NA	61.3%	57.0%	61.8%	63.4%	60.7%	<0.001
% Aware of HIV+ status (among those testing HIV+)	28.8%	39.2%	45.7%	30.4%	35.4%	36.1%	51.1%	56.7%	69.6%	63.6%	75.7%	65.3%	<0.001
% Registered in AIDS centre (of self‐reported HIV+)	84.2%	77.1%	76.4%	84.9%	80.4%	79.9%	88.7%	84.5%	92.6%	94.0%	93.3%	91.3%	<0.001
% On ART (of those self‐reported HIV+)	NA	27.7%	49.1%	51.5%	57.0%	45.5%	NA	32.8%	57.6%	66.7%	73.3%	59.5%	<0.001
% HIV+	20.0%	19.7%	14.4%	18.0%	17.8%	17.8%	30.4%	28.8%	24.1%	33.3%	31.2%	29.2%	<0.001
% HIV+ of PWID aged < 25 years	6.9%	6.2%	3.5%	3.9%	3.3%	4.9%	16.5%	13.8%	6.3%	6.7%	5.9%	10.5%	<0.001
% HCV Ab+	NA	32.9%	50.3%	50.1%	57.8%	48.1%	NA	47.7%	66.7%	66.2%	75.4%	64.5%	<0.001
% HCV Ab+ of PWID aged < 25 years	NA	16.2%	29.3%	22.2%	25.2%	22.8%	NA	33.1%	41.4%	35.0%	44.1%	37.8%	<0.001

ART, antiretroviral therapy; HCV, hepatitis C virus; OAT, opiate agonist therapy; PWID, people who inject drugs.

^a^ χ^2^ test for binary variables, or a t‐test for continuous variables, stratified by non‐NGO versus NGO client status across the combined survey year groups

^b^ among those who had had sex

^c^ composite variable formed of the above two variables.

### 3.2 Characteristics associated with being an NGO client

Table 3 shows unadjusted and adjusted odds ratios of being an NGO client for various demographic characteristics. PWID testing HIV+ and HCV+ were more likely to be NGO clients. PWID that were female, that had ever been imprisoned, that were registered in a drug abuse clinic, or had higher education were more likely to be NGO clients, whereas PWID released from prison within the last 12 months were less likely to be NGO clients. In the sensitivity analysis (Table S5) removing the ever imprisoned and HCV variables and including the 2009 survey data, produced similar results.

**Table 3:** Unadjusted and adjusted odds ratios (with 95% confidence intervals) from mixed‐effect logistic regression^a^, of being an NGO client for various demographic characteristics

Variable	Unadjusted OR (95% CI)	*p*‐value	Adjusted OR (95% CI) [N = 37,845]	*p*‐value
HIV+ [N = 38,053]	2.18 (2.06, 2.30)	<0.001	1.48 (1.39, 1.57)	<0.001
Hepatitis C virus (HCV) antibody+ [N = 38,052]	2.30 (2.19, 2.42)	<0.001	1.72 (1.63, 1.81)	<0.001
Age (years) [N = 38,053]	1.03 (1.02, 1.03)	<0.001	1.00 (1.00, 1.01)	0.151
Female [N = 37,920]	1.32 (1.25, 1.40)	<0.001	1.43 (1.35, 1.51)	<0.001
Ever imprisoned [N = 37,917]	1.68 (1.61, 1.76)	<0.001	1.28 (1.21, 1.36)	<0.001
Imprisoned in the last 12 months [N = 38,053]	0.93 (0.84, 1.02)	0.116	0.66 (0.59, 0.73)	<0.001
Registered in a drug abuse clinic [N = 38,053]	3.24 (3.08, 3.41)	<0.001	2.61 (2.48, 2.76)	<0.001
Education [N = 37,848]				
Primary education	1		1	
Incomplete secondary education	0.87 (0.75, 1.01)	0.062	0.82 (0.70, 0.95)	0.011
Complete secondary education	0.85 (0.74, 0.98)	0.024	0.81 (0.70, 0.94)	0.006
Basic higher education	0.92 (0.79, 1.07)	0.260	0.94 (0.80, 1.10)	0.415
Complete higher education	0.93 (0.79, 1.09)	0.376	0.98 (0.83, 1.16)	0.845

CI, confidence interval; OR, odds ratio.

^a^ With survey year and city as the crossed random effects – 2009 was excluded for this analysis due to missing data for HCV and ever imprisoned.

### 3.3 Associations between being an NGO client and intervention‐related outcomes

**Figure 1:**
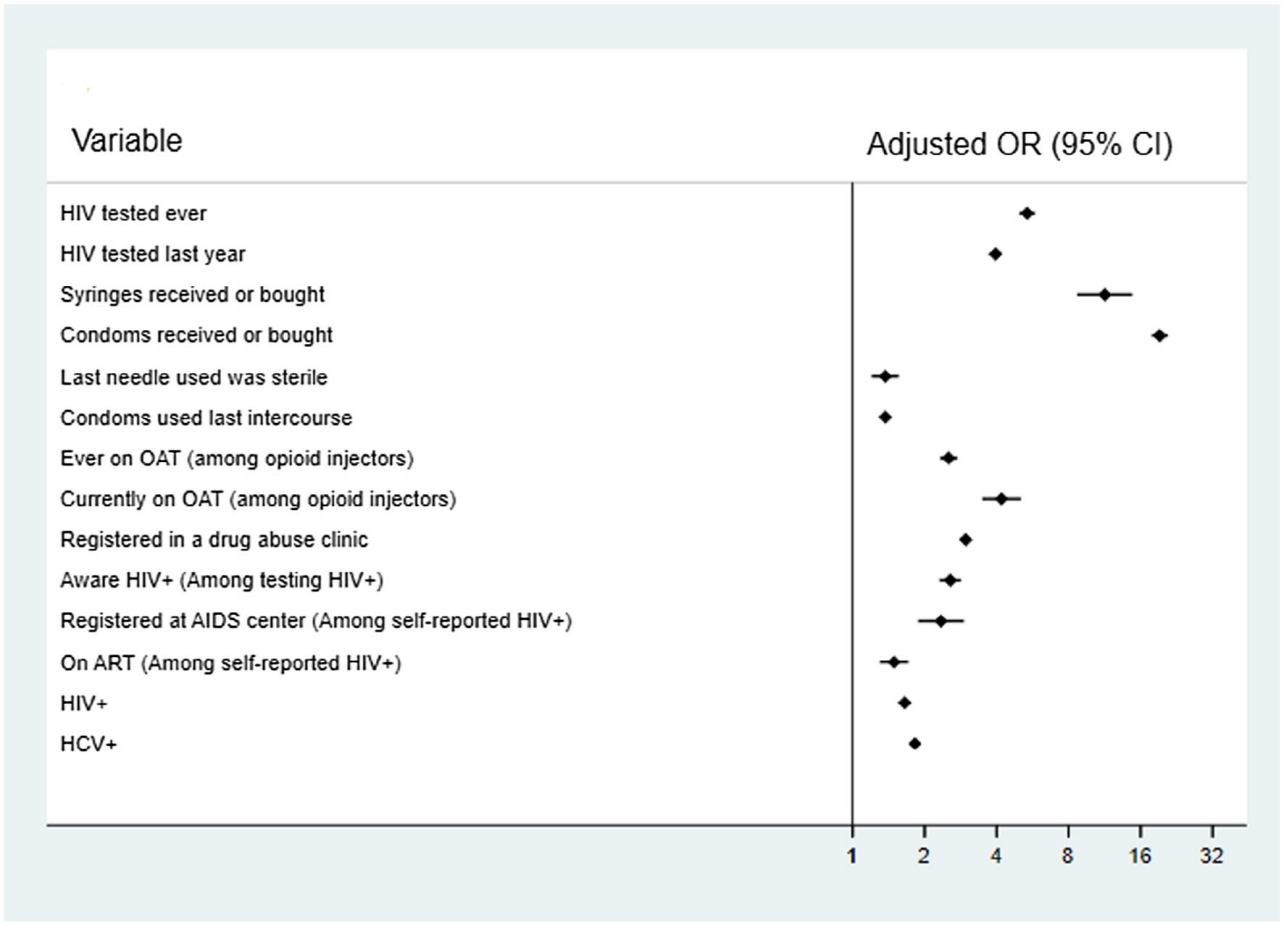
Adjusted* odds ratios (with 95% confidence intervals) for various outcomes from being an NGO client compared to not being an NGO client, using mixed‐effect logistic regression. *Adjusted for age, gender, whether they had ever been imprisoned, whether they are registered at a drug clinic, and education level. Models have year and city as mixed‐effects except for the HCV model that only has year as a mixed‐effect. The model with being registered in a drug abuse clinic as an outcome did not adjust for this variable.

The differences between NGO and non‐NGO clients seen in Table 2 are borne out in Table 4 and Figure 1. In both unadjusted and adjusted regression analyses, NGO clients were more likely to have ever been tested for HIV, to have been tested in the last year, to have used a sterile needle for their last injection, to have used a condom for their last intercourse, to currently or have ever been on OAT, to be registered in a drug abuse clinic, to be registered at an AIDS centre, and to self‐report being on ART (among those self‐reporting as HIV+), and to test HIV+ or HCV+. NGO clients were more likely than non‐clients to have received syringes and condoms in the last year, but were less likely to have bought syringes or condoms in the last month. When combining these measures, NGO clients were more likely to have received or bought syringes, with the same result for condoms. Among the HIV‐negative PWID, NGO clients were more likely to have been tested for HIV in the last year (adjusted odds ratio: 3.96, 95% confidence interval: 3.73 to 4.19) than non‐NGO clients.

**Table 4:** Unadjusted and adjusted odds ratios (with 95% confidence intervals) from mixed‐effect logistic regression for various intervention related outcomes from being an NGO client compared to not being an NGO client

Virus and harm reduction‐related outcomes	Unadjusted OR (95% CI)	Adjusted OR (95% CI)^b^					N
	NGO client	NGO client	Age (years)	Female (vs. male)	Ever imprisoned (vs. not)	Registered in drug abuse clinic	
HIV tested ever	6.79 (6.30, 7.32)	5.37 (4.97, 5.80)	1.01 (1.01, 1.02)	1.47 (1.37, 1.57)	1.68 (1.58, 1.78)	3.04 (2.83, 3.26)	37,845
HIV tested last year	3.87 (3.68, 4.06)	3.37 (3.20, 3.54)	0.98 (0.98, 0.99)	1.24 (1.17, 1.31)	1.17 (1.11, 1.23)	1.86 (1.77, 1.96)	37,845
Syringes received last year	121.83 (110.54, 134.27)	109.89 (99.26, 121.66)	1.00 (1.00, 1.01)	1.26 (1.17, 1.36)	1.12 (1.04, 1.20)	2.14 (2.00, 2.29)	37,845
Bought syringes last month	0.13 (0.12, 0.14)	0.12 (0.11, 0.13)	0.97 (0.97, 0.97)	0.64 (0.60, 0.68)	1.37 (1.28, 1.46)	1.04 (0.98, 1.11)	37,845
Received or bought syringes^d^	52.91 (41.62, 67.26)	11.33 (8.69, 14.77)	0.98 (0.97, 0.99)	0.48 (0.43, 0.55)	1.38 (1.19, 1.59)	1.75 (1.49, 2.06)	37,845
Condoms received last year	51.81 (48.13, 55.76)	54.39 (50.17, 58.96)	0.98 (0.98, 0.99)	1.10 (1.02, 1.18)	1.06 (0.99, 1.14)	1.92 (1.80, 2.05)	37,845
Bought condoms last month	0.22 (0.21, 0.24)	0.25 (0.23, 0.27)	0.95 (0.95, 0.95)	0.51 (0.48, 0.55)	0.91 (0.85, 0.96)	0.88 (0.83, 0.94)	37,845
Received or bought condoms^d^	18.38 (17.13, 19.72)	19.24 (17.80, 20.80)	0.96 (0.96, 0.96)	0.67 (0.63, 0.71)	0.94 (0.89, 0.99)	1.40 (1.32, 1.49)	37.845
Last needle used was sterile	1.46 (1.29, 1.65)	1.37 (1.20, 1.56)	0.99 (0.98, 1.00)	0.98 (0.86, 1.12)	0.92 (0.82, 1.04)	1.00 (0.88, 1.13)	37.845
Condoms used last intercourse (among those who had had sex)	1.29 (1.23, 1.35)	1.37 (1.30, 1.44)	0.97 (0.97, 0.98)	0.66 (0.63, 0.70)	0.75 (0.71, 0.79)	1.19 (1.12, 1.25)	32,726
Ever on OAT (among primary opioid injectors)	3.87 (3.58, 4.19)	2.52 (2.32, 2.74)	1.01 (1.00, 1.01)	1.09 (0.99, 1.20)	1.36 (1.25, 1.48)	6.51 (5.95, 7.13)	30,649
Currently on OAT (among primary opioid injectors)	7.14 (6.00, 8.49)	4.19 (3.48, 5.05)	1.01 (1.00, 1.02)	1.27 (1.05, 1.54)	1.11 (0.93, 1.32)	12.29 (9.75, 15.49)	15,807
Registered in a drug abuse clinic	3.25 (3.10, 3.41)	2.97 (2.82, 3.13)	1.04 (1.03, 1.04)	0.98 (0.92, 1.04)	2.48 (2.35, 2.61)	NA	37,845
Aware of HIV+ status (among those testing HIV+)	3.09 (2.81, 3.39)	2.56 (2.31, 2.84)	1.02 (1.01, 1.02)	1.52 (1.36, 1.70)	1.38 (1.24, 1.53)	2.14 (1.93, 2.37)	8061
Registered at AIDS centre (among those self‐reporting HIV+)	3.05 (2.46, 3.77)	2.34 (1.88, 2.92)	1.05 (1.03, 1.07)	1.90 (1.47, 2.46)	0.92 (0.73, 1.16)	3.56 (2.83, 4.47)	4302
On ART (among those self‐reporting HIV+)	1.60 (1.40, 1.83)	1.49 (1.30, 1.71)	1.05 (1.04, 1.06)	1.06 (0.91, 1.23)	1.02 (0.88, 1.18)	1.28 (1.11, 1.47)	4286
HIV+	2.18 (2.06, 2.31)	1.65 (1.56, 1.75)	1.04 (1.03, 1.04)	1.72 (1.62, 1.84)	1.80 (1.70, 1.91)	2.03 (1.91, 2.15)	37,845
HCV Ab+	2.30 (2.19, 2.42)	1.82 (1.73, 1.92)	1.03 (1.02, 1.03)	1.03 (0.97, 1.09)	1.82 (1.73, 1.91)	2.03 (1.93, 2.15)	37,845

^a^ Mixed‐effect logistic regression models with year and city as crossed mixed‐effects. Survey data from 2009 were omitted as data on imprisonment was missing for this round

^b^ only year was included as a mixed‐effect variable in this model due to problems with model fitting

^c^ Table S6 gives the adjusted odds ratios for education categories

^d^ composite variable created from the two above.

### 3.4 NGO client duration

Trends in characteristics and behaviours by NGO client duration (per year increase among NGO clients) are shown in Table 5, with PWID that have been NGO clients for longer tending to be older, more likely to have ever been imprisoned or tested for HIV, and less likely to have bought syringes or condoms in the last month. The more experienced NGO clients were also more likely to be registered in a drug abuse clinic, to have ever been on OAT or to currently be on OAT and were more likely to be HIV+ or HCV+. Among those self‐reporting as HIV+, the longer someone was an NGO client the more likely they were to be registered at an AIDS centre or to be on ART.

**Table 5:** Outcomes linked to bloodborne viruses and their transmission, stratified by duration as a client of a non‐governmental organization (NGO), with a test for trend by duration of NGO contact (per year increase)^a^

Outcome	NGO client duration				OR (95% CI) for those with known duration^a^	Not NGO client
	0 to 2 years	3 to 5 years	6+ years	Unknown		
	[N = 5495]	[N = 3651]	[N = 1747]	[N = 1581]		[N = 25,579]
Age (years)	33.3	35.5	38.4	34.7	1.86 (1.77, 1.96)	33.6
Ever imprisoned	40.1%	46.3%	58.7%	45.2%	1.04 (1.03, 1.06)	33.7%
Syringes received year	95.5%	96.4%	94.4%	92.9%	0.98 (0.94, 1.01)	20.4%
Condoms received year	92.1%	92.6%	89.2%	87.9%	0.98 (0.96, 1.01)	19.6%
Bought syringes last year	51.7%	45.4%	53.0%	61.9%	0.98 (0.96, 0.99)	88.4%
Bought condoms last year	9.7%	7.3%	7.0%	5.0%	0.96 (0.93, 0.99)	25.4%
Last needle sterile	97.1%	97.9%	97.0%	95.5%	1.00 (0.95, 1.04)	95.4%
Condom last intercourse (among those who had had sex)	58.0%	57.7%	51.1%	54.6%	0.99 (0.98, 1.01)	48.9%
HIV test ever	90.5%	95.7%	97.5%	88.8%	1.22 (1.17, 1.27)	64.8%
HIV test last year	62.0%	56.8%	61.2%	65.0%	1.00 (0.99, 1.02)	29.5%
Ever OAT (Among primary opioid injectors)	17.3%	22.5%	29.5%	15.9%	1.09 (1.07, 1.11)	6.5%
Current OAT (Among primary opioid injectors)	10.5%	12.2%	15.3%	8.2%	1.08 (1.05, 1.11)	2.0%
Registered in a drug abuse clinic	42.2%	54.5%	65.3%	47.4%	1.12 (1.10, 1.14)	24.5%
Aware of HIV+ status (among those testing HIV+)	60.5%	69.0%	81.5%	60.8%	1.10 (1.07, 1.13)	37.0%
Registered at AIDS centre (of self‐reported HIV+)	89.1%	91.9%	95.8%	89.8%	1.20 (1.10, 1.29)	79.5%
On ART (of self‐report HIV+)	54.2%	58.2%	71.4%	62.2%	1.05 (1.01, 1.08)	46.0%
HIV+	25.7%	29.4%	38.4%	29.2%	1.07 (1.05, 1.08)	17.5%
HCV+	62.0%	64.0%	73.7%	63.8%	1.03 (1.01, 1.05)	48.1%

ART, antiretroviral therapy; CI, confidence intervals; HCV, hepatitis C virus; OAT, opiate agonist therapy; OR, odds ratio.

^a^ Tests performed using mixed‐effects logistic regression modelling (linear for age as the outcome) among PWID that have a known duration as an NGO client. This NGO client duration is included as an independent variable and year and city included as crossed mixed‐effects respectively. Models are also adjusted for age, except for the model with age as the outcome. Data from 2009 were omitted as information on NGO client duration was missing, whereas only data were available for 2015 and 2017 for the current OAT outcome.

### 3.5 Use of services by PWID

**Figure 2:**
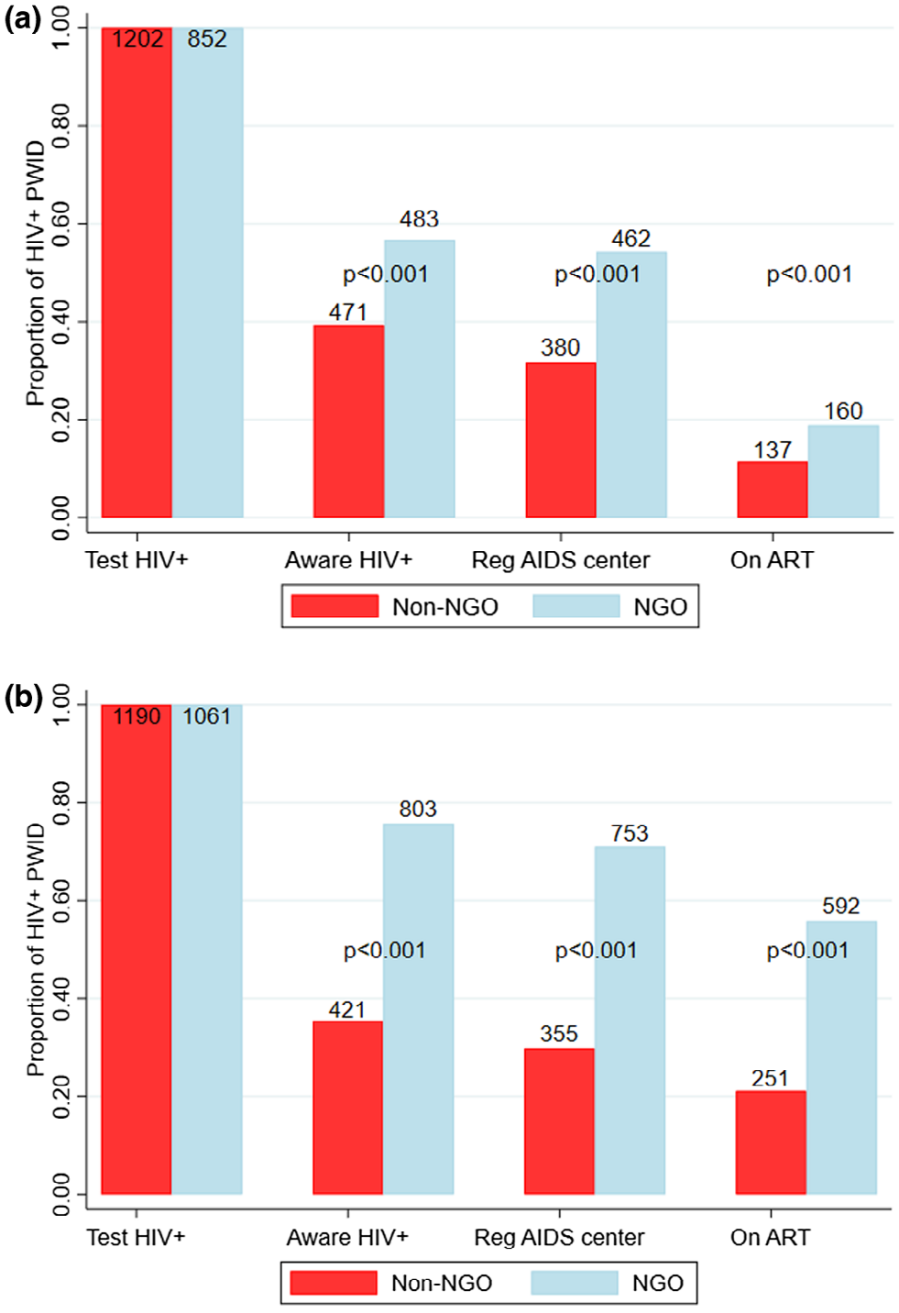
Self‐reported use of HIV services by HIV‐positive PWID for 2011 (left) and 2017 (right), stratified by whether they are NGO clients or not, with tests for differences in proportions across groups. ART, antiretroviral therapy. NGO, non‐governmental organization; Reg, Registered at; SR, self‐report.

Figure 2 shows the self‐reported use of HIV services in 2011 and 2017 for those testing HIV+. For both 2011 and 2017, better outcomes were seen for each outcome among NGO clients (*p* < 0.001). A higher proportion of NGO clients reported being aware of their HIV+ status, more were registered at an AIDS centre, and more self‐reported being on ART. The disparities between the outcomes for the NGO and non‐NGO clients increased from 2011 to 2017.

## 4 DISCUSSION

In this analysis, data from five IBBS in Ukraine spanning 2009 to 2017 showed that PWID in contact with harm reduction NGOs obtained more condoms and new injecting equipment, were more likely to use sterile needles and condoms, and were more likely to be on OAT. These better outcomes were seen despite NGO clients being more marginalized in terms of ever being imprisoned, being poorer, and injecting more frequently. They were also more likely to be HCV positive or HIV positive, with HIV‐positive NGO clients being more engaged in using HIV services than non‐NGO clients. Many of these associations became stronger with longer NGO client duration, which could be due to a dose–response relationship or possibly survivor bias – further research on this topic is required.

Data suggested the use of services by PWID improved over time, with the percentage of PWID ever tested for HIV increasing by half and the percentage of HIV‐positive PWID on ART doubling from 2009/2011 to 2017. The differences in outcomes between NGO and non‐NGO clients also increased between 2011 and 2017, possibly as NGOs became more effective. These results should be considered in light of increases in ART coverage over this time, making interpretation of this trend complicated.

### 4.1 Comparison with other literature

Other studies in Ukraine have considered the HIV cascade of care and prevalence trends. An analysis of 2015 IBBS data among HIV‐positive PWID also found evidence that NGO‐status was positively associated with self‐reported registration at an AIDS centre and receiving ART [22]. Otherwise, a study from 2011 to 2014 using intervention monitoring and evaluation data found that harm reduction coverage (receiving more syringes and condoms) was associated with decreased HIV acquisition risk [23], adding to our findings that harm reduction coverage is linked to improvements in risk behaviours and coverage of OAT and ART.

Notably, considering the high imprisonment rates among PWID in this analysis, other studies in Ukraine have highlighted the importance of the prison setting in driving the HIV and HCV epidemics [24, 25, 26], emphasizing the importance of controlling for imprisonment whenever possible. A modelling study found that scaling up OAT in Ukraine from the current levels (approximately 2.7% of PWID) to 20% would reduce new HIV infections by 56% and deaths by 49% over 10 years [27]. Lastly, few studies have focussed on HCV in Ukraine, with two finding similarly high HCV prevalence among PWID (approximately 60%) [28, 29].

### 4.2 Strengths and limitations

The strengths of our analyses include using multiple national level IBBS with large sample sizes, spanning many years that ask comparable questions over the surveys. The questions asked spanned diverse topics allowing examination of many outcomes, with our findings consistently suggesting that harm reduction contact has multiple benefits. Importantly, the use of a national IBBS also means the results should be generalizable to the national level.

While most questions were the same or similar across years, some questions changed or were unavailable for particular years, especially 2009. Also, while most cities/regions were sampled in all IBBS, some were omitted for certain rounds. HIV viral load was only tested among a subsample of the HIV‐positive PWID (to help estimate HIV incidence) in later surveys so could not be used as a biological marker of successful ART uptake. The analyses were limited to looking at associations rather than causation. While the results for HIV and HCV infection exposure were based on biological testing, all other behaviours and uptake of interventions were based on self‐reported data. These could be affected by a range of biases, such as recall bias, particularly as many questions ask about behaviours over a long timeframe. Social desirability bias could lead to an overestimation of harm reduction practices and an underestimation of risky practices. Despite this, it is uncertain whether there would be differential bias between NGO clients and non‐clients. Another potential limitation is that RDS was used to enrol participants and sample characteristics may change with each survey, although our results are similar with and without RDS weighting. Additionally, the quality of data captured may have improved with each round of the survey due to an increase in expertise of groups (often NGOs) carrying out the survey, which frequently stayed the same across years. However, once again, it remains unclear whether this would differentially affect NGO and non‐NGO clients. There is also the possibility that the question used to determine our main analysis measure, whether or not someone was a client of an NGO, could have been misunderstood by some participants, with some having had contact with NGOs but not being clients. This may mean our results are conservative because NGOs may also have positive outcomes among non‐clients. Carrying out some of the surveys in the offices of organizations that provide services for PWID could also have created information bias where PWID were aware that those carrying out the surveys were linked to NGOs and so preferred to report behaviours that were deemed desirable to the interviewers. Unfortunately, data on survey setting are not available so we could not examine this effect.

## 5 CONCLUSIONS

Ukraine has the second largest HIV and HCV epidemics in Europe [7, 8], for which IDU drives transmission [3, 4]. NGOs are a major provider of harm reduction services (including needles, syringes and condoms) and testing of HIV and HCV for PWID in Ukraine, whereas the government provides OAT and ART. As of November 2019, support for core NGO services will transition to government funding, with the Global Fund only supporting extended prevention programmes, such as naloxone. Our findings suggest harm reduction NGOs are benefiting PWID in Ukraine by improving access to needles and condoms, increasing OAT uptake, and improving all aspects of the HIV continuum of care. Getting PWID onto ART is crucial for halting the Ukrainian HIV epidemic, whereas harm reduction interventions are crucial for reducing HIV and HCV transmission [10, 30]. For Ukraine to meet the UN’s 90‐90‐90 HIV goals and elimination targets [31] or WHO’s HCV elimination targets [32], government policy‐makers need to ensure sufficient funding continues for these interventions since resources from the Global Fund have decreased [15].

The reduction in Global Fund support for harm reduction NGOs comes during uncertain times for Ukraine. The country has recently emerged from a recession and is still engaged in a war with Russia, resulting in population migration [33], including those with HIV and HCV [34]. This situation is exacerbated by the ongoing stigma borne by both PWID and people living with HIV [35, 36]. Additionally, the ongoing COVID‐19 pandemic will likely cause huge disruption to the Ukrainian economy [37]. Against this backdrop of competing priorities for the Ukrainian government, it is important that funding for harm reduction programmes is not reduced, as they are associated with public health benefits [10, 23, 30, 38] and have been shown to be cost‐effective [39, 40, 41]. Indeed, an economic evaluation from 2018 suggested the role of NGOs should be expanded to provide HIV care and treatment [41], something that will start in October 2020 through support from the US Centres for Disease Control and Prevention. It is important that these services continue and are expanded because if funding decreases, the HIV and HCV epidemics among PWID could escalate to higher incidence [42], with further transmission bridging to other population groups.

## COMPETING INTEREST

All authors have contributed to the study and/or manuscript and provided their approval to submit. NS, TS, YS and OV work for the Alliance for Public Health (APH), Ukraine, which is a non‐governmental organization. APH is one of the largest recipients in Ukraine of funding from the Global Fund to fight AIDS, tuberculosis, and malaria (GF), and salaries of YS and TS are funded through GF grants. JS reports non‐financial support from Gilead Sciences, outside the submitted work. JGW reports previous grants from CDC Foundation and a current research grant from Gilead unrelated to this work. PV reports grants from National Institute of Drug Abuse and National Institute of Health Research. PV has received unrestricted research grants from Gilead unrelated to this work.

## AUTHORS’ CONTRIBUTIONS

AT performed the analyses and wrote the first draft of the report, with guidance from PV, AGL, JGW and JS. PV had the original idea for the study. NS, TS, YS and OV contributed to data collection, data cleaning, data interpretation and of writing of the report. All authors have read and approved the final manuscript.

## INFORMED CONSENT

Informed consent was obtained from all study participants.

## Supporting information


**Table S1.** A tabulation of the number of survey respondents by year and city
**Table S2.** Variables by survey question
**Table S3.** Behaviours and preventive outcomes among PWID across each survey year using respondent‐driven sampling (RDS) weighting
**Table S4.** Education‐level stratified by year and whether the PWID are clients of an NGO
**Table S5.** Unadjusted and adjusted odds ratios (with 95% confidence intervals) from mixed‐effect logistic regression*, of being an NGO client for various demographic characteristics – sensitivity analysis of Table 3 removing the ever imprisoned and HCV variables and therefore including the 2009 survey data
**Table S6.** Adjusted odds ratios (with 95% confidence intervals) from mixed‐effect logistic regression for education categories on various intervention related outcomes (corresponding to Table 4)
**Table S7.** Residual intraclass correlation for each outcome*

## References

[1] Degenhardt L , Peacock A , Colledge S , Leung J , Grebely J , Vickerman P , et al. Global prevalence of injecting drug use and sociodemographic characteristics and prevalence of HIV, HBV, and HCV in people who inject drugs: a multistage systematic review. Lancet Glob Health. 2017;5(12):E1192–207. 10.1016/S2214-109X(17)30375-3 PMC5683738 29074409

[2] Grebely J , Larney S , Peacock A , Colledge S , Leung J , Hickman M , et al. Global, regional, and country‐level estimates of hepatitis C infection among people who have recently injected drugs. Addiction. 2019;114(1):150–66. 30035835 10.1111/add.14393 PMC6657799

[3] Degenhardt L , Charlson F , Stanaway J , Lamey S , Alexander LT , Hickman M , et al. Estimating the burden of disease attributable to injecting drug use as a risk factor for HIV, hepatitis C, and hepatitis B: findings from the Global Burden of Disease Study 2013. Lancet Infect Dis. 2016;16(12):1385–98. 27665254 10.1016/S1473-3099(16)30325-5

[4] Trickey A , Fraser H , Lim AG . The contribution of injection drug use to hepatitis C virus transmission globally, regionally, and at country level: a modelling study. Lancet Gastroenterol. 2019;4(6):435‐44. 10.1016/S2468-1253(19)30085-8 PMC6698583 30981685

[5] UNAIDS . Global AIDS update 2016. 2016.

[6] Blach S , Zeuzem S , Manns M , Altraif I , Duberg AS , Muljono DH , et al. Global prevalence and genotype distribution of hepatitis C virus infection in 2015: a modelling study. Lancet Gastroenterol. 2017;2(3):161–76. 10.1016/S2468-1253(16)30181-9 28404132

[7] ECDC . HIV, AIDS surveillance in Europe. 2018.

[8] Maistat L , Kravchenko N , Reddy A . Hepatitis C in Eastern Europe and Central Asia: a survey of epidemiology, treatment access and civil society activity in eleven countries. Hepatol Med Policy. 2017;2:9. 30288322 10.1186/s41124-017-0026-z PMC6171005

[9] Mukandavire C , Low A , Mburu G , Trickey A , May MT , Davies CF , et al. Impact of opioid substitution therapy on the HIV prevention benefit of antiretroviral therapy for people who inject drugs. Aids. 2017;31(8):1181–90. 28323752 10.1097/QAD.0000000000001458

[10] Platt L , Minozzi S , Reed J , Vickerman P , Hagan H , French C , et al. Needle and syringe programmes and opioid substitution therapy for preventing HCV transmission among people who inject drugs: findings from a Cochrane Review and meta‐analysis. Addiction. 2018;113(3):545–63. 28891267 10.1111/add.14012 PMC5836947

[11] Trickey A , Fraser H , Lim AG , Walker JG , Peacock A , Colledge S , et al. Modelling the potential prevention benefits of a treat‐all hepatitis C treatment strategy at global, regional and country levels: a modelling study. J Viral Hepatitis. 2019;26(12):1388–403. 10.1111/jvh.13187 PMC10401696 31392812

[12] Cohen MS , Chen YQ , McCauley M , Gamble T , Hosseinipour MC , Kumarasamy N , et al. Antiretroviral therapy for the prevention of HIV‐1 transmission. New Engl J Med. 2016;375(9):830–9. 27424812 10.1056/NEJMoa1600693 PMC5049503

[13] UNAIDS . Prevention gap report. 2016.

[14] The Global Fund . Global fund grants to Ukraine. Geneva, Switzerland: The Global Fund; 2018.

[15] PEPFAR . Ukraine Country Operational Plan (COP) 2018 ‐ strategic direction summary. 2018.

[16] Clark D . Ukraine’s economy has turned a corner. The Financial Times. 2017.

[17] Alliance for Public Health . Behavior monitoring and HIV‐infection prevalence among injection drug users. 2010.

[18] Alliance for Public Health . Behaviour monitoring and HIV‐prevalence among injecting drug users as a component of second generation surveillance. 2012.

[19] Alliance for Public Health . Summary of the analytical report: Monitoring the behaviour and HIV‐infectino prevalence among people who inject drugs as a component of HIV second generation surveillance. 2014.

[20] Alliance for Public Health . Monitoring of behavior and HIV prevalence among people who inject drugs and their sexual partners. 2016.

[21] Avery L , Rotondi N , McKnight C , Firestone M , Smylie J , Rotondi M . Unweighted regression models perform better than weighted regression techniques for respondent‐driven sampling data: results from a simulation study. BMC Med Res Methodol. 2019;19(1):202. 31664912 10.1186/s12874-019-0842-5 PMC6819607

[22] Dumchev K , Varetska O , Salyuk T , Vitek C . HIV treatment cascade analysis for people who inject drugs in Ukraine: identifying the correlates of HIV care outcomes. Journal of International AIDS. Society. 2017.

[23] Ompad DC , Wang J , Dumchev K , Barska J , Samko M , Zeziulin O , et al. Patterns of harm reduction service utilization and HIV incidence among people who inject drugs in Ukraine: a two‐part latent profile analysis. Int J Drug Policy. 2017;43:7–15. 28160736 10.1016/j.drugpo.2016.12.008 PMC6714049

[24] Altice FL , Azbel L , Stone J , Brooks‐Pollock E , Smyrnov P , Dvoriak S , et al. The perfect storm: incarceration and the high‐risk environment perpetuating transmission of HIV, hepatitis C virus, and tuberculosis in Eastern Europe and Central Asia. Lancet. 2016;388(10050):1228–48. 27427455 10.1016/S0140-6736(16)30856-X PMC5087988

[25] Azbel L , Wickersham JA , Grishaev Y , Dvoryak S , Altice FL . Burden of infectious diseases, substance use disorders, and mental illness among Ukrainian prisoners transitioning to the community. PLoS One. 2013;8:e59643. 23527238 10.1371/journal.pone.0059643 PMC3602355

[26] Csete J , Kamarulzaman A , Kazatchkine M , Altice F , Balicki M , Buxton J , et al. Public health and international drug policy. Lancet. 2016;387(10026):1427–80. 27021149 10.1016/S0140-6736(16)00619-X PMC5042332

[27] Tan J , Altice FL , Madden LM , Zelenev A . Effect of expanding opioid agonist therapies on the HIV epidemic and mortality in Ukraine: a modelling study. Lancet HIV. 2020;7(2):e121–e8. 10.1016/S2352-3018(19)30373-X PMC7289179 31879250

[28] Iakunchykova O , Meteliuk A , Zelenev A , Mazhnaya A , Tracy M , Altice FL . Hepatitis C virus status awareness and test results confirmation among people who inject drugs in Ukraine. Int J Drug Policy. 2018;57:11–7. 29655101 10.1016/j.drugpo.2018.03.022 PMC5994183

[29] Zelenev A , Shea P , Mazhnaya A , Meteliuk A , Pykalo I , Marcus R , et al. Estimating HIV and HCV prevalence among people who inject drugs in 5 Ukrainian cities using stratification‐based respondent driven and random sampling. Int J Drug Policy. 2019;67:91–101. 30503695 10.1016/j.drugpo.2018.09.010 PMC6537868

[30] MacArthur GJ , Minozzi S , Martin N , Vickerman P , Deren S , Bruneau J , et al. Opiate substitution treatment and HIV transmission in people who inject drugs: systematic review and meta‐analysis. BMJ. 2012;345:e5945. 23038795 10.1136/bmj.e5945 PMC3489107

[31] UNAIDS . 90–90‐90 An ambitious treatment target to help end the AIDS epidemic. 2017.

[32] World Health Organization . Combating hepatitis B and C to reach elimination by 2030. Geneva: World Health Organization; 2016 https://www.who.int/hepatitis/publications/hep-elimination-by-2030-brief/en/

[33] Holt E . The alliance for public health, Ukraine. Lancet HIV. 2018;5(6):e276. 10.1016/S2352-3018(18)30108-5 29893242

[34] Vasylyeva TI , Liulchuk M , Friedman SR , Sazonova I , Faria NR , Katzourakis A , et al. Molecular epidemiology reveals the role of war in the spread of HIV in Ukraine. Proc Natl Acad Sci USA. 2018;115(5):1051–6. 29339468 10.1073/pnas.1701447115 PMC5798316

[35] Biancarelli DL , Biello KB , Childs E , Drainoni M , Salhaney P , Edeza A , et al. Strategies used by people who inject drugs to avoid stigma in healthcare settings. Drug Alcohol Depen. 2019;198:80–6. 10.1016/j.drugalcdep.2019.01.037 PMC6521691 30884432

[36] Turan B , Budhwani H , Fazeli PL , Browning WR , Raper JL , Mugavero MJ , et al. How does stigma affect people living with HIV? The mediating roles of internalized and anticipated hiv stigma in the effects of perceived community stigma on health and psychosocial outcomes. AIDS Behav. 2017;21(1):283–91. 27272742 10.1007/s10461-016-1451-5 PMC5143223

[37] Organisation for Economic Co‐operation and Development . The COVID‐19 crisis in Ukraine. 2020.

[38] Cepeda JA , Eritsyan K , Vickerman P , Lyubimova A , Shegay M , Odinokova V , et al. Potential impact of implementing and scaling up harm reduction and antiretroviral therapy on HIV prevalence and mortality and overdose deaths among people who inject drugs in two Russian cities: a modelling study. Lancet HIV. 2018;5(10):E578–87. 10.1016/S2352-3018(18)30168-1 PMC6188805 30033374

[39] Vickerman P , Kumaranayake L , Balakireva O , Guinness L , Artyukh O , Semikop T , et al. The cost‐effectiveness of expanding harm reduction activities for injecting drug users in Odessa, Ukraine. Sex Transm Dis. 2006;33 10 Suppl:S89–102. 16735956 10.1097/01.olq.0000221335.80508.fa

[40] Sweeney S , Ward Z , Platt L , Guinness L , Hickman M , Hope V , et al. Evaluating the cost‐effectiveness of existing needle and syringe programmes in preventing hepatitis C transmission in people who inject drugs. Addiction. 2019;114(3):560–70. 30674091 10.1111/add.14519

[41] Deloitte Latypov A , Dierst‐Davies R , Sereda Y , Kerr CC , Duda M , et al.HIV investment case study for Ukraine: Evaluation of program costs, service quality, and resource allocation for HIV expenditure in 2015. 2018.

[42] Booth RE , Davis JM , Dvoryak S , Brewster JT , Lisovska O , Strathdee SA , et al. HIV incidence among people who inject drugs (PWIDs) in Ukraine: results from a clustered randomised trial. Lancet HIV. 2016;3(10):E482–9. 10.1016/S2352-3018(16)30040-6 PMC5101021 27658879

