# Supplementary material for: Has resourcing of non‐governmental harm‐reduction organizations in Ukraine improved HIV prevention and treatment outcomes for people who inject drugs? Findings from multiple bio‐behavioural surveys
Source: J Int AIDS Soc. 2020 Aug 26;23(8):e25608. doi: 10.1002/jia2.25608 (PMC7450208; doi:10.1002/jia2.25608)
Supplement: Supplementary file 1 — Table S1. A tabulation of the number of survey respondents by year and city Table S2. Variables by survey question Table S3. Behaviours and preventive outcomes among PWID across each survey year using respondent‐driven sampling (RDS) weighting Table S4. Education‐level stratified by year and whether the PWID are clients of an NGO Table S5. Unadjusted and adjusted odds ratios (with 95% confidence intervals) from mixed‐effect logistic regression*, of being an NGO client for various demographic characteristics – sensitivity analysis of Table 3 removing the ever imprisoned and HCV variables and therefore including the 2009 survey data Table S6. Adjusted odds ratios (with 95% confidence intervals) from mixed‐effect logistic regression for education categories on various intervention related outcomes (corresponding to Table 4) Table S7. Residual intraclass correlation for each outcome* [file JIA2-23-e25608-s001.docx]

**Supplementary materials**

**Supplementary table 1:** A tabulation of the number of survey respondents by year and city.

| **City** | **Region** | **2009** | **2011** | **2013** | **2015** | **2017** | **Total** |
| --- | --- | --- | --- | --- | --- | --- | --- |
| Simferopol | Crimea | 252 | 500 | 401 | 400 | 0 | 1,553 |
| Vinnitsya | Vinnitsya | 250 | 350 | 250 | 250 | 250 | 1,350 |
| Lutsk | Volyn | 0 | 352 | 350 | 350 | 250 | 1,302 |
| Dnipro | Dnipropetrovsk | 249 | 499 | 501 | 500 | 548 | 2,297 |
| Donetsk | Donetsk | 0 | 501 | 500 | 444 | 507 | 1,952 |
| Zhytomyr | Zhytomyr | 249 | 350 | 350 | 350 | 420 | 1,719 |
| Uzhgorod | Zakarpattia | 100 | 200 | 200 | 150 | 200 | 850 |
| Zaporizhzhya | Zaporizhzhya | 249 | 200 | 350 | 150 | 250 | 1,199 |
| Ivano-Frankivsk | Ivano-Frankivsk | 250 | 250 | 300 | 350 | 300 | 1,450 |
| Bila Tserkva | Kyiv | 0 | 299 | 300 | 350 | 350 | 1,299 |
| Kyiv | Kyiv city | 407 | 508 | 500 | 399 | 350 | 2,164 |
| Kropyvnytskyi | Kirovohrad | 0 | 350 | 250 | 300 | 250 | 1,150 |
| Severodonetsk | Luhansk | 253 | 251 | 300 | 150 | 250 | 1,204 |
| Lviv | Lviv | 249 | 250 | 350 | 401 | 403 | 1,653 |
| Mykolaiv | Mykolaiv | 250 | 500 | 500 | 500 | 500 | 2,250 |
| Odesa | Odesa | 0 | 500 | 400 | 450 | 400 | 1,750 |
| Poltava | Poltava | 0 | 350 | 300 | 150 | 401 | 1,201 |
| Rivne | Rivne | 254 | 350 | 300 | 400 | 299 | 1,603 |
| Sumy | Sumy | 0 | 350 | 350 | 150 | 200 | 1,050 |
| Ternopil | Ternopil | 101 | 200 | 250 | 350 | 248 | 1,149 |
| Kharkiv | Kharkiv | 0 | 353 | 350 | 200 | 300 | 1,203 |
| Kherson | Kherson | 0 | 351 | 300 | 400 | 350 | 1,401 |
| Khmelnytskyi | Khmelnytskyi | 0 | 350 | 300 | 450 | 250 | 1,350 |
| Cherkasy | Cherkasy | 249 | 356 | 350 | 350 | 450 | 1,755 |
| Chernivtsi | Chernivtsi | 101 | 200 | 250 | 150 | 250 | 951 |
| Chernigiv | Chernigiv | 250 | 349 | 300 | 360 | 400 | 1,659 |
| Sevastopol | Sevastopol city | 0 | 0 | 350 | 401 | 350 | 1,101 |
| Vasylkiv | Kyiv | 0 | 0 | 150 | 150 | 300 | 600 |
| Fastive | Kyiv | 0 | 0 | 150 | 400 | 250 | 800 |
| Kryvyi Rig | Dnipropetrovsk | 249 | 0 | 0 | 0 | 550 | 799 |
| Melitopol | Zaporizhzhya | 0 | 0 | 0 | 0 | 250 | 250 |
|  | Total: | 3,962 | 9,069 | 9,502 | 9,405 | 10,076 | 42,014 |

**Supplementary table 2:** Variables by survey question.

| **Variable** | **Survey question** |
| --- | --- |
| Education | What is your educational level? 1. Primary education (9 incomplete years of school); 2. Basic (incomplete) secondary education (9 complete years of school); 3. Complete secondary education (or professional-technical education) (11 years of school), incomplete higher education; 4. Basic higher education (higher education establishments of I-II accreditation levels, technical colleague); 5. Complete higher education (bachelor, master) (higher education establishments of III-IV accreditation levels), (university, institute); 6. Other (specify) |
| Age of first injection | How old were you when you used (injecting) drugs for the first time |
| Injections last month | How often have you used injecting drugs in the last month |
| Primary drug is opioid | Which of the injecting drugs do you consider a primary one for you? |
| Overdosed last year | Have you had overdoses in the last 12 months? (The 2009 questionnaire asks about opiate overdoses) |
| Ever on OAT | Have you ever received methadone or buprenorphine in the program of substitution maintenance therapy (SMT)? |
| Currently on OAT | Are you currently receiving the methadone or buprenorphine in the program of substitution maintenance therapy (SMT)? |
| Registered in drug abuse clinic | Please say if you are registered in a state drug abuse clinic because of injection drug use? |
| Currently homeless | What has been your permanent place of residence in the last 3 months? Option: Street, abandoned apartments, railway stations (homeless). |
| Ever imprisoned | Have you ever served sentence? |
| Imprisoned in last year | When were you released from prison last time? |
| Last needle used was sterile | The last time you injected drugs, did you use a sterile needle and syringe? |
| Needles used last month that were unsterile | In the last 30 days, did you inject a drug with a syringe previously used by another person? |
| Using a pre-filled syringe last month | Have you received/bought an injection from an already filled syringe in the last 30 days? |
| Using condom last intercourse | Did you (or your partner) use a condom during the last sexual intercourse? |
| Received syringes last year | Have you received sterile syringes/needles in the last 12 months (for example, from members or volunteers of informational and educational programmes or projects, syringe exchange sites etc.)? |
| Buying syringes last month | Have you bought syringes/needles for yourself in the last month? |
| Received or bought syringes | No question available: this is a composite measure of the two variables above. |
| Received condoms last year | Have you received condoms in the last 12 months (for example, through informational and educatinoal programmes or projects, syringe exchange sites, consultation centers, centers for social services for family, children and youth, during actions etc.)? |
| Buying condoms last month | Have you bought condoms for yourself in the last month? |
| Received or bought condoms | No question available: this is a composite measure of the two variables above. |
| HIV tested ever | I am not asking now about the test result, but have you ever had an HIV-test? |
| HIV tested last year | …was it within the last 12 months? |
| Aware of HIV+ status | Do you agree to tell us your HIV-status? If yes, was it positive/negative. |
| Registered in AIDS center | Please say if you are registered in the AIDS center. |
| On ART | Are you on antiretroviral therapy (ART)? |

*Trends over time*

The number of PWID surveyed in each IBBS were 3,963 (2009), 9,069 (2011), 9,502 (2013), 9,405 (2015), and 10,076 (2017). Table 1 shows characteristics and behaviours of the PWID surveyed in each year and tests for trends. Across all surveys, around one-third of PWID surveyed self-reported as NGO clients, which was stable over the years. The percentage of PWID that were female decreased from 23.4% in 2009 to 17.8% in 2017, whilst the mean age increased from 30.7 years to 35.5 years. HIV prevalence was stable over the surveys (~21.5%) but decreased among those aged <25 years from 9.0% in 2009 to 3.7% in 2017. The HCV prevalence among all PWID increased from 37.7% in 2011 to 63.8% in 2017 but remained stable among PWID aged <25 years (~26.0%). The percentage of PWID reporting ever being on OAT was stable, whilst the percentage ever tested for HIV increased from 50.0% to 78.5%. Although the proportion of self-reported HIV-positive participants registered at AIDS centers remained stable (~86.6%), the percentage of those registered at AIDS centers who self-reported being on ART doubled from 30.3% in 2011 to 67.5% in 2017. The percentage that overdosed in the previous year decreased from 12.9% to 5.2% over 2009-2017. The percentage reporting current homelessness was low (<0.5%). The percentage of PWID that received syringes last year decreased from 51.6% to 39.1%, as did the percentage receiving condoms from 47.8% to 37.3%. Meanwhile, the percentage buying syringes in the last month increased from 67.1% to 84.7%, whilst the percentage buying condoms in the last month was stable. The percentage either buying or receiving syringes remained stable (~96%), whilst the percentage either buying or receiving condoms decreased from 65.7% to 52.5%. Using a sterile needle at the time of last injection increased over the years, whilst condom use at last intercourse decreased.

**Supplementary table 3:** Behaviours and preventive outcomes among PWID across each survey year using respondent-driven sampling (RDS) weighting.

| **Variable** | **2009** | **2011** | **2013** | **2015*** | **2017** |
| --- | --- | --- | --- | --- | --- |
| % NGO client | 28.2% | 27.7% | 34.7% | 23.7% | 30.9% |
| Mean NGO client duration (years) | NA | 2.4 | 2.7 | 2.8 | 4.9 |
| % Female | 24.5% | 26.3% | 23.2% | 20.4% | 18.1% |
| % Completed secondary education | 82.4% | 83.8% | 78.8% | 82.0% | 81.6% |
| Mean age (years) | 30.1 | 33.4 | 34.2 | 34.9 | 36.0 |
| Mean age of first injection (years) | 20.0 | 20.6 | 20.4 | 20.7 | 20.9 |
| Mean injecting duration (years) | 10.1 | 12.8 | 13.8 | 14.2 | 15.5 |
| Mean injections last month | NA | 22.2 | 15.0 | 17.4 | 20.2 |
| % Primary drug is opioid | 79.5% | 77.6% | 82.0% | 80.1% | 81.9% |
| % Overdosed last year | 13.4% | 7.0% | 6.0% | 5.0% | 4.9% |
| % Ever on OAT | NA | 6.8% | 11.5% | 12.9% | 10.8% |
| % Currently on OAT | NA | NA | NA | 4.4% | 4.8% |
| % Registered in drug abuse clinic | 26.6% | 30.1% | 31.7% | 30.0% | 29.8% |
| % Currently homeless | NA | NA | 0.5% | 0.3% | 0.2% |
| % Ever imprisoned | NA | 33.4% | 34.1% | 38.0% | 41.1% |
| % Imprisoned in last year | NA | 5.1% | 4.9% | 9.8% | 5.1% |
| % Last needle used was sterile | 86.9% | 95.4% | 96.7% | 94.1% | 97.1% |
| % Needles used last month that were unsterile | NA | 2.3% | 1.4% | 1.1% | 0.8% |
| % Using a pre-filled syringe last month | 55.1% | 58.6% | 53.7% | 44.9% | 31.6% |
| % Using condom last intercourse (among those who had had sex) | 54.6% | 54.8% | 54.0% | 50.9% | 47.1% |
| % Received syringes last year | 39.3% | 42.3% | 46.2% | 35.4% | 36.8% |
| % Buying syringes last month | NA | 66.9% | 68.0% | 80.6% | 82.6% |
| % Received or bought syringes** | NA | 94.8% | 96.6% | 94.2% | 96.6% |
| % Received condoms last year | 36.7% | 42.5% | 44.2% | 33.2% | 35.4% |
| % Buying condoms last month | NA | 23.6% | 22.7% | 21.6% | 18.9% |
| % Received or bought condoms** | NA | 62.1% | 62.5% | 50.6% | 51.4% |
| % HIV tested ever | 38.4% | 64.7% | 72.9% | 70.5% | 77.8% |
| % HIV tested last year | NA | 37.6% | 40.3% | 37.2% | 39.8% |
| % Aware of HIV+ status (among those testing HIV+) | 26.3% | 47.2% | 62.5% | 46.3% | 57.3% |
| % Registered in an AIDS center (of those self-reported HIV+) | 92.2% | 84.1% | 71.6% | 94.3% | 93.6% |
| % On ART (of those registered in an AIDS center) | NA | 31.8% | 41.1% | 65.4% | 71.5% |
| % HIV+ | 20.9% | 22.3% | 18.9% | 21.5% | 22.1% |
| % HIV+ of PWID aged<25 years | 8.5% | 8.0% | 4.6% | 5.0% | 2.5% |
| % HCV+ | NA | 35.4% | 54.0% | 51.6% | 62.7% |
| % HCV+ of PWID aged<25 years | NA | 15.9% | 28.6% | 21.3% | 26.5% |

*24 duplicate RDS IDs dropped; ** Composite variable created from the above two variables. PWID: People who inject drugs. NGO: Non-governmental organisation. OAT: Opiate agonist therapy. ART: Antiretroviral therapy. HCV: Hepatitis C virus.

**Supplementary table 4:** Education-level stratified by year and whether the PWID are clients of an NGO.

|  | **2009** | **2011** | **2013** | **2015** | **2017** |
| --- | --- | --- | --- | --- | --- |
| **Education** | **Non-NGO** | |  |  |  |
| Primary | 3.3% | 2.3% | 3.1% | 3.6% | 2.1% |
| Incomplete secondary | 14.2% | 14.4% | 16.5% | 14.6% | 15.5% |
| Complete secondary | 67.1% | 59.6% | 60.3% | 61.8% | 62.7% |
| Basic higher | 10.7% | 15.8% | 14.0% | 11.3% | 11.2% |
| Complete higher | 4.8% | 7.9% | 6.2% | 8.8% | 8.5% |
|  | **NGO** |  |  |  |  |
| Primary | 3.3% | 2.2% | 4.2% | 2.2% | 1.9% |
| Incomplete secondary | 16.9% | 10.4% | 18.8% | 12.6% | 13.9% |
| Complete secondary | 61.7% | 56.0% | 54.6% | 64.0% | 64.6% |
| Basic higher | 12.9% | 21.5% | 15.7% | 13.1% | 11.3% |
| Complete higher | 5.3% | 9.9% | 6.7% | 8.2% | 8.4% |
| χ^2^ test for differences | 0.012 | <0.001 | <0.001 | <0.001 | 0.229 |

**Supplementary table 5:** Unadjusted and adjusted odds ratios (with 95% confidence intervals) from mixed-effect logistic regression*, of being an NGO client for various demographic characteristics – sensitivity analysis of table 3 removing the ever imprisoned and HCV variables and therefore including the 2009 survey data.

| **Variable** | **Unadjusted OR (95% CI)** | **P-value** | **Adjusted OR (95% CI)**  **[N=41,798]** | **P-value** |
| --- | --- | --- | --- | --- |
| HIV+ | 1.94 (1.85, 2.04) | <0.001 | 1.63 (1.54, 1.72) | <0.001 |
| Age (years) | 1.02 (1.02, 1.02) | <0.001 | 1.01 (1.01, 1.01) | <0.001 |
| Female | 1.36 (1.29, 1.43) | <0.001 | 1.34 (1.27, 1.41) | <0.001 |
| Registered in a drug abuse clinic | 3.06 (2.93, 3.19) | <0.001 | 2.93 (2.78, 3.07) | <0.001 |
|  |  |  |  |  |
| Education |  |  |  |  |
| Primary education | 1 |  | 1 |  |
| Incomplete secondary education | 0.87 (0.76, 1.01) | 0.065 | 0.83 (0.72, 0.96) | 0.014 |
| Complete secondary education | 0.85 (0.75, 0.97) | 0.019 | 0.81 (0.71, 0.93) | 0.003 |
| Basic higher education | 0.90 (0.78, 1.04) | 0.148 | 0.89 (0.77, 1.03) | 0.119 |
| Complete higher education | 0.93 (0.80, 1.09) | 0.380 | 0.95 (0.81, 1.11) | 0.485 |

OR: Odds ratio. CI: Confidence interval.

*With survey year and city as the crossed random effects.

**Supplementary table 6**: Adjusted odds ratios (with 95% confidence intervals) from mixed-effect logistic regression for education categories on various intervention related outcomes (corresponding to table 4).

|  | **Education category** | | | | |
| --- | --- | --- | --- | --- | --- |
| **Virus and harm reduction-related outcomes** | **Primary** | **Basic (incomplete) secondary** | **Complete secondary or incomplete higher** | **Basic higher** | **Complete higher** |
| HIV tested ever | 1 | 1.28 (1.09, 1.50) | 1.55 (1.33, 1.80) | 1.62 (1.37, 1.90) | 1.86 (1.56, 2.21) |
| HIV tested last year | 1 | 0.96 (0.83, 1.12) | 1.09 (0.95, 1.25) | 1.10 (0.94, 1.27) | 1.26 (1.08, 1.48) |
| Syringes received last year | 1 | 0.80 (0.66, 0.97) | 0.84 (0.70, 1.01) | 0.95 (0.78, 1.15) | 0.88 (0.71, 1.09) |
| Bought syringes last month | 1 | 1.23 (1.02, 1.47) | 1.42 (1.20, 1.68) | 1.26 (1.06, 1.52) | 1.65 (1.36, 2.01) |
| Received or bought syringes* | 1 | 1.06 (0.73, 1.55) | 1.25 (0.88, 1.78) | 1.19 (0.81, 1.74) | 1.30 (0.86, 1.95) |
| Condoms received last year | 1 | 0.94 (0.78, 1.14) | 1.01 (0.84, 1.21) | 1.11 (0.91, 1.35) | 0.99 (0.80, 1.21) |
| Bought condoms last month | 1 | 1.11 (0.93, 1.33) | 1.30 (1.10, 1.54) | 1.47 (1.23, 1.76) | 1.52 (1.25, 1.83) |
| Received or bought condoms* | 1 | 0.99 (0.85, 1.17) | 1.15 (0.99, 1.34) | 1.38 (1.17, 1.62) | 1.27 (1.07, 1.50) |
| Last needle used was sterile | 1 | 1.07 (0.79, 1.46) | 1.45 (1.08, 1.94) | 1.39 (1.01, 1.92) | 1.43 (1.01, 2.02) |
| Condoms used last intercourse (among those who had had sex) | 1 | 1.06 (0.91, 1.24) | 1.14 (0.99, 1.32) | 1.28 (1.10, 1.50) | 1.15 (0.97, 1.35) |
| Ever on OAT (among primary opioid injectors) | 1 | 0.91 (0.71, 1.16) | 0.92 (0.73, 1.16) | 0.92 (0.71, 1.18) | 1.40 (1.08, 1.82) |
| Currently on OAT (among primary opioid injectors) | 1 | 1.06 (0.64, 1.76) | 1.11 (0.68, 1.79) | 1.00 (0.59, 1.72) | 1.55 (0.91, 2.65) |
| Registered in a drug abuse clinic | 1 | 1.10 (0.94, 1.29) | 1.16 (1.00, 1.35) | 1.16 (0.99, 1.37) | 1.09 (0.92, 1.30) |
| Aware of HIV+ status (Among those testing HIV+) | 1 | 1.13 (0.84, 1.53) | 1.17 (0.87, 1.55) | 0.99 (0.72, 1.36) | 1.04 (0.73, 1.46) |
| Registered at AIDS center (Among those self-reporting HIV+) | 1 | 0.91 (0.46, 1.81) | 0.96 (0.50, 1.84) | 1.07 (0.51, 2.21) | 1.39 (0.61, 3.15) |
| On ART (Among those self-reporting HIV+) | 1 | 0.82 (0.53, 1.27) | 0.93 (0.61, 1.40) | 0.87, (0.55, 1.36) | 0.97 (0.60, 1.59) |
| HIV+ | 1 | 1.06 (0.89, 1.25) | 0.98 (0.84, 1.15) | 0.78 (0.66, 0.94) | 0.73 (0.60, 0.88) |
| HCV Ab+ | 1 | 1.17 (1.01, 1.36) | 1.16 (1.01, 1.33) | 1.08 (0.93, 1.25) | 1.13 (0.97, 1.32) |

*Composite variable formed from the two variables above.

Education categories: 1. Primary education (9 incomplete years of school); 2. Basic (incomplete) secondary education (9 complete years of school); 3. Complete secondary education (or professional-technical education) (11 years of school), incomplete higher education; 4. Basic higher education (higher education establishments of I-II accreditation levels, technical colleague); 5. Complete higher education (bachelor, master) (higher education establishments of III-IV accreditation levels), (university, institute).

**Supplementary table 7: Residual intraclass correlation for each outcome***

| **Virus and harm reduction-related outcomes** | **City** | **Year** |
| --- | --- | --- |
| HIV tested ever | 0.01 (0.00, 0.07) | 0.11 (0.09, 0.15) |
| HIV tested last year | 0.02 (0.01, 0.06) | 0.09 (0.07, 0.12) |
| Syringes received last year | 0.06 (0.02, 0.15) | 0.24 (0.19, 0.30) |
| Bought syringes last month | 0.05 (0.02, 0.15) | 0.25 (0.20, 0.31) |
| Received or bought syringes* | 0.04 (0.01, 0.14) | 0.21 (0.16, 0.27) |
| Condoms received last year | 0.03 (0.01, 0.11) | 0.19 (0.15, 0.23) |
| Bought condoms last month | 0.02 (0.01, 0.05) | 0.07 (0.05, 0.10) |
| Received or bought condoms* | 0.02 (0.01, 0.07) | 0.11 (0.08, 0.14) |
| Last needle used was sterile | 0.01 (0.00, 0.12) | 0.08 (0.06, 0.11) |
| Condoms used last intercourse (among those who had had sex) | 0.02 (0.01, 0.04) | 0.05 (0.04, 0.07) |
| Ever on OAT (among primary opioid injectors) | 0.09 (0.04, 0.17) | 0.22 (0.17, 0.29) |
| Currently on OAT (among primary opioid injectors) | 0.14 (0.04, 0.41) | 0.36 (0.26, 0.49) |
| Registered in a drug abuse clinic | 0.09 (0.05, 0.15) | 0.16 (0.11, 0.21) |
| Aware of HIV+ status (Among those testing HIV+) | 0.02 (0.00, 0.11) | 0.15 (0.11, 0.20) |
| Registered at AIDS center (Among those self-reporting HIV+) | 0.07 (0.02, 0.21) | 0.21 (0.14, 0.31) |
| On ART (Among those self-reporting HIV+) | 0.00 (0.00, 0.00) | 0.15 (0.11, 0.21) |
| HIV+ | 0.14 (0.08, 0.22) | 0.18 (0.12, 0.25) |
| HCV Ab+ | 0.06 (0.02, 0.13) | 0.20 (0.16, 0.25) |

*Taken from mixed-effects models with year nested within city.
